# Social autopsy: INDEPTH Network experiences of utility, process, practices, and challenges in investigating causes and contributors to mortality

**DOI:** 10.1186/1478-7954-9-44

**Published:** 2011-08-05

**Authors:** Karin Källander, Daniel Kadobera, Thomas N Williams, Rikke Thoft Nielsen, Lucy Yevoo, Aloysius Mutebi, Jonas Akpakli, Clement Narh, Margaret Gyapong, Alberta Amu, Peter Waiswa

**Affiliations:** 1 Department of Health Policy, Planning & Management, School of Public Health, Makerere University, P.O. Box 7072, Kampala, Uganda; 2 Iganga/Mayuge Health & Demographic Surveillance Site (HDSS), P.O. Box 111, Iganga, Uganda; 3 Department of Public Health Sciences, Division of International Health (IHCAR), Nobels Väg 9, Karolinska Institutet, Stockholm 17176, Sweden; 4 Malaria Consortium Africa, P.O box 8045, Kampala, Uganda; 5 KEMRI-Wellcome Trust Research Programme, Epidemiological and Demographic Surveillance System (EPI-DSS) Group, Kilifi, Kenya; 6 Nuffield Department of Clinical Medicine, Centre for Tropical Medicine, University of Oxford, Churchill Hospital, Old Road, Oxford OX3 7LJ, UK; 7 Bandim Health Project, Apartado 861, Bissau, 1004 Bissau Codex, Guinea-Bissau; 8 Statens Serum Institut, 5 Artillerivej, Copenhagen 2300, Denmark; 9 Dodowa Health Research Centre, Ghana Health Service, P.O. Box 1, Dodowa, Ghana

## Abstract

**Background:**

Effective implementation of child survival interventions depends on improved understanding of cultural, social, and health system factors affecting utilization of health care. Never the less, no standardized instrument exists for collecting and interpreting information on how to avert death and improve the implementation of child survival interventions.

**Objective:**

To describe the methodology, development, and first results of a standard social autopsy tool for the collection of information to understand common barriers to health care, risky behaviors, and missed opportunities for health intervention in deceased children under 5 years old.

**Methods:**

Under the INDEPTH Network, a social autopsy working group was formed to reach consensus around a standard social autopsy tool for neonatal and child death. The details around 434 child deaths in Iganga/Mayuge Health and Demographic Surveillance Site (HDSS) in Uganda and 40 child deaths in Dodowa HDSS in Ghana were investigated over 12 to 18 months. Interviews with the caretakers of these children elicited information on what happened before death, including signs and symptoms, contact with health services, details on treatments, and details of doctors. These social autopsies were used to assess the contributions of delays in care seeking and case management to the childhood deaths.

**Results:**

At least one severe symptom had been recognized prior to death in 96% of the children in Iganga/Mayuge HDSS and in 70% in Dodowa HDSS, yet 32% and 80% of children were first treated at home, respectively. Twenty percent of children in Iganga/Mayuge HDSS and 13% of children in Dodowa HDSS were never taken for care outside the home. In both countries most went to private providers. In Iganga/Mayuge HDSS the main delays were caused by inadequate case management by the health provider, while in Dodowa HDSS the main delays were in the home.

**Conclusion:**

While delay at home was a main obstacle to prompt and appropriate treatment in Dodowa HDSS, there were severe challenges to prompt and adequate case management in the health system in both study sites in Ghana and Uganda. Meanwhile, caretaker awareness of danger signs needs to improve in both countries to promote early care seeking and to reduce the number of children needing referral. Social autopsy methods can improve this understanding, which can assist health planners to prioritize scarce resources appropriately.

## Background

Each year approximately 8.8 million deaths occur in children younger than 5 years old worldwide, with the majority (68%) dying of infectious causes such as pneumonia, diarrhea, and malaria [1]. Reliable estimates of the numbers, causes of, and contributors to death are key elements of functional health systems where health policies and programs are evidence based [2]. However, especially in high mortality settings, the civil registration systems are limited or nonexistent, and most deaths go unrecorded [3]. Fewer than one-third of the 57 million global annual deaths are issued medical certificates [4]. Countries that cannot record the number of people who die or why they die cannot realize the full potential of their health systems [5]. As an alternative method, the estimation of cause-specific mortality can be obtained through the use of alternative methods, such as verbal autopsies (VAs), which use standard interview tools with caretakers on symptoms preceding death in a patient [6,7].

Most of the INDEPTH coordinated Health and Demographic Surveillance Sites (HDSS) (http://www.indepth-network.org), which carry out longitudinal surveillance of births, deaths, and migrations in defined populations, have already adopted VA for routine investigation of cause of death. These data are used to generate area-specific disease profiles, which are shared with subnational and national health planners for better allocation of health resources. However, these VA tools do not provide information on critical delays and care seeking that could have saved the child.

Several previous attempts have been made to understand the reasons why diseases like malaria, diarrhea, and pneumonia continue to cause so many child deaths. The contributing factors leading to death are complex, but poor recognition of illness symptoms by parents and inappropriate medical care provided to children appears to play an important role in many countries [8-20]. Better information about the social processes, the timing and type of care-seeking actions, and treatments received prior to death is critical to identifying modifiable factors that can be addressed by new policies or better resource planning. In this paper we suggest bringing these methodologies together under one standard umbrella called social autopsy (SA).

This paper presents the consensus products and results of a three-year effort by an expert group of researchers, pediatricians, statisticians, and other stakeholders under the sponsorship of the INDEPTH Network. It is intended to serve the needs of various users and producers of mortality information, including researchers, policymakers, program managers, and evaluators in the 37 INDEPTH field sites in 19 countries. To make these resources as easily and widely accessible as possible, they will be published on the INDEPTH website. The expert group on social autopsy systematically reviewed, debated, and refined the accumulated experience and evidence from the most widely-used social autopsy questionnaires and procedures. This resulted in standard social autopsy questionnaires for two age groups (neonatal and child deaths). The results from applying the child death SA tool in Uganda and Ghana is presented, and points of intervention that could, in the future, prevent other deaths are identified and discussed. The neonatal tool was piloted in Guinea-Bissau and in Uganda, and the results will be presented elsewhere.

## Methods

### Study area and population

#### Uganda

The Iganga/Mayuge HDSS is a defined area across parts of Iganga and Mayuge districts at the shores of Lake Victoria in eastern Uganda. The HDSS area is predominantly rural, but partly peri-urban in some trading areas. It consists of 185 villages drawn from both Iganga and Mayuge districts. It spans a total area of 400 km^2 ^with a total population of approximately 159,000. The main source of income is subsistence farming with a culturally homogenous population (more than 80% are Lusoga speaking). About 17% are children less than 5 years old. The overall under-5 mortality is 137 deaths per 1,000 live births [21], and the key causes of child mortality are neonatal conditions (24%), malaria (23%), pneumonia (21%), and diarrheal diseases (17%) [22]. The Iganga/Mayuge HDSS has one hospital, eight public health centers, three non-governmental organization (NGO) clinics, and 122 drug shops. The site is a member of the international HDSS organization INDEPTH and largely follows its standard methods.

#### Ghana

The Dodowa HDSS is housed within the Dodowa Health Research Centre. Studies conducted in the center have a focus on developing and evaluating community- and district-based health interventions and obtaining information to improve health policy, planning, and service delivery in the Ghana Health Service. It has strong links with the District Health Management Team and the Local Government Authority. The HDSS is sited in Dodowa, the district capital of the Dangme West District of the Greater Accra Region. It covers a population of approximately 98,000 people in 381 communities. It is purely rural but gradually catching up with the rapid urbanization of the peripheral areas surrounding the city of Accra. The most common form of transportation in the district is the bicycle. Under-5 mortality in Ghana is 80 deaths per 1,000 live births in the most recent five-year period [23], and the top causes are similar to those in Uganda, namely neonatal conditions (28%), malaria (33%), pneumonia (15%), and diarrheal diseases (12%) [24]. The Dangme West District has four health centers and six community clinics spread throughout the district. In the traditional sector, there are 300 traditional healers, 92 trained traditional birth attendants (TBAs) and "wanzams" (local circumcisers), and an equal number of untrained TBAs who provide alternative medical services. In addition, there are approximately 58 chemical sellers and an unknown number of drug peddlers operating throughout the district. The district has no hospitals. The inhabitants use surrounding hospitals for referral care as well as for some primary care. The same hospitals are the referral hospitals for the community health insurance scheme, which operates in the district.

### Sampling

The HDSSs in Uganda and Ghana generate population-based data on key demographic events two to three times a year and household, socioeconomic, and education data annually or biannually. In Iganga/Mayuge HDSS, deaths are identified either by information obtained from the routinely (every six months) updated demographic population registry, or through reporting by the one or two village scouts who are selected in each village to report all births and deaths in their respective villages in the study area. In Dodowa HDSS, deaths are identified from three sources: 1) a list from the Dodowa HDSS Field Office based on information collected from the routinely updated demographic population registry, 2) deaths reported from the health facilities, and 3) deaths reported from community key informants.

In Iganga/Mayuge HDSS all reported child deaths from January 2009 to July 2010 were included in the study (n = 434). In Dodowa HDSS it was found after the pilot that the community key informants only concentrated on some of the communities and, since the routine population registry is only updated every 6 months, many child deaths were missed by the system. Hence, only 40 child deaths were reported between December 2008 and December 2009, and all of these were included in the study.

### Data collection

For all deaths in the Iganga/Mayuge and Dodowa HDSSs, verbal autopsies are used to assign likely cause of death. In Iganga/Mayuge HDSS in Uganda, a modified version of the social autopsy questionnaire developed in Bolivia [25] has been integrated into the standardized verbal autopsy tool and used routinely since 2006. In December 2008, both Iganga/Mayuge and Dodowa HDSS introduced the modified social autopsy tools developed by the social autopsy working group (SAWG). In Dodowa, the SA tools were integrated into the verbal autopsy tool four months after its introduction (April 2009). Whenever a child death was reported, after a mourning period of four to six weeks, the merged VA/SA questionnaire was used by a trained native field worker fluent in the local language (four in Dodowa and 12 in Iganga/Mayuge) to interview the deceased's immediate care giver about symptoms presented, preventive behavior, treatment-seeking behavior, and detailed information about care received by first and last provider visited.

### Instruments

Through Internet-based searches using Medline and Google free-text search and communications with researchers known to have experience with death inquiry methodology, published and unpublished reports were retrieved and reviewed for good and bad practices in using postmortem questionnaires to collect data on care-seeking practices prior to death. A total of nine questionnaires were retrieved and reviewed, of which one had been used in Guinea-Bissau, one in India, two in Kenya, four in Uganda, and one in Bolivia. Observations from the review included the inability of previously-used tools to assess the timing of treatment seeking in relation to the severity of symptoms, the sequence of the many providers visited, and the quality of care provided. The results of the review were compiled and discussed in the SAWG, a group consisting of medical doctors, social scientists, epidemiologists, demographers, and statisticians from INDEPTH HDSSs in Guinea-Bissau, Uganda, Kenya, and Ghana. The group reached consensus on the content of the tools that would result in a minimum level of complexity during data collection and analysis.

Based on the review findings, it was decided that there was a need for specific tools for neonatal (0 to 28 days old) and under-5 (29 days old to 5 years old) death events. The new SA tool for under-5 deaths incorporated learning from the mortality surveys in Bolivia [20], where the analysis of the care-seeking process for all providers seen was found to be too complex. Hence, the new SA tool was designed to capture only information on the first and last provider seen before death.

The new tool also begins with a history section with probes for the respondents to describe recognition of symptoms, timing of recognition, actions taken at home and outside, and provider behavior. Other detailed information collected in the new tool includes:

• Symptoms presented (e.g., symptoms in chronological order starting with day 1, time from first symptom to death, and in relation to provider seen)

• Preventive behaviour (e.g., bed net use, vaccinations)

• Treatment-seeking behavior (e.g., type, place, and timing of treatment at home and outside the home; chronological order of providers seen; reasons for not giving treatment or seeking care; timing and sequence of providers seen; transport used for seeking care; cost of transport, treatment, and care; hospitalization of child; and reason for and compliance with referral)

The new SA tool for under-5 deaths was piloted for 12 to 18 months for all deaths in these age groups in Dodowa HDSS in Ghana and in Iganga/Mayuge HDSS in Uganda, and the results from data collection were entered and analyzed. Bandim HDSS in Guinea-Bissau only piloted the newborn SA tool, and the results will be presented elsewhere.

### Data analysis

Data were entered in FoxPro (Microsoft Corporation, Seattle, WA, USA) and analyzed in STATA 10 (Stata Corporation, College Station, TX, USA).

The BASICS conceptual framework [26], which suggests a number of indicators that can be quantified for standardized analysis and comparability over time and space, was adopted for analysis of care-seeking processes preceding death. The data were subjected to standard descriptive analysis using proportions (overall and conditional) for categorical data (e.g., children with severe symptoms treated at home) and median values with interquartile range for continuous data (e.g., length of illness before death). Numbers were inserted into an Excel spreadsheet that listed all indicators and was programmed to automatically yield proportions, conditional proportions, and graphical outputs (available on request from first author).

Caretaker *recognition *of severe illness was defined by the caretakers' mention of at least one of the danger signs defined by the Integrated Management of Childhood Illness (IMCI) guidelines [27]. These include convulsions, chest indrawing, nasal flaring, grunting, bulging fontanelle, umbilical redness extending to the skin, many or severe skin pustules, lethargic or unconscious or less than normal movement, not able to drink or breastfeed, vomits everything, convulsions, loose stools/diarrhea > 2 days, heavy bleeding

Similarly, "possibly severe" illness symptoms (wheezing, fever, fast breathing, and difficult breathing) were also retrieved from the IMCI guidelines. *Informal care *was defined as any care provider that was not a public/private health facility, hospital, or trained community health worker (CHW). *Severity of illness *at the time of care seeking was not explored in the version of the SA tool that was piloted in this study but was later added to the revised version to capture this potential source of bias.

A modified version of Thaddeus' and Maine's three-delay model for maternal death [28] was adapted, as described by Waiswa et al. 2010 [11], for determining the occurrence of delays in the home, on the way to, and in the health facility. The definitions of delay at the different levels are provided in Tables 1 and 2. *Delay in the home *included lack of recognition of at least one severe symptom; treatment of children recognized to have at least one possibly severe or severe symptom at home; treatment at home without going for any outside care; treatment of children with severe signs who were not taken for outside care on the same day or with possibly severe symptoms who were not taken for outside care after 24 hours; treatment from an informal care provider as both first and last source; or not complying with referral advice because of reasons such as waiting to get permission from husband, belief that the child was too sick to go, waiting to finish ongoing treatment, or other answers related to perception of illness.

**Table 1:** Three-delay model for child deaths in Uganda

Delay clusters	Number of children	Denominator	Conditional proportion	**Relative weight**^**¤**^	Cluster total weight*	**Proportional contribution (total = 0.70)**^**∞**^
**Delay 1 - home delay**				**1/6**	**0.17**	**24%**

# children whose caregivers did not mention at least one severe symptom	17	f	4%	0.01		

# children with possibly severe or severe symptom who were treated at home	126	d	32%	0.05		

# children only receiving treatment at home without going outside for care	85	f	20%	0.03		

# children with severe symptoms who were brought outside the home for care after > 1 day	174	c	42%	0.07		

# children who only received informal health care for their fatal illnesses as both first and last source of care	3	b	1%	0.00		

# not going for referral because of caretaker decision-making	6	e	4%	0.01		

**Delay 2 - transport delay**				**1/2**	**0.22**	**32%**

# delaying > 2 hrs to reach first or last provider	84	a	36%	0.18		

# not going for referral because of lack of money for transport	17	e	12%	0.04		

**Delay 3 - health facility delay**				**1/3**	**0.31**	**44%**

# children obtaining treatment from provider after > 1 hr from first or last provider	71	a	20%	0.07		

# children referred because of lack of equipment or lack of drugs	92	e	65%	0.22		

# deceased children who did not receive any treatment after visiting first or last formal provider	17	b	7%	0.02		

**# who went to at least one outside provider (a)**	349					

**# who went to at more than one outside provider (b)**	234					

**# children reported with at least one severe symptom (c)**	417					

**# children reported with at least one severe or possibly severe symptom (d)**	398					

**# children referred from first or last provider (e)**	141					

**Total # child deaths (f)**	434					

^¤ ^Each indicator's relative contribution to the delay within a cluster, assuming each indicator is equally important.

*The total contribution to delay of each cluster (total = 0.70)

**^∞ ^**The proportional contribution to delay of each cluster.

**Table 2:** Three-delay model for child deaths in Ghana

	Number of children	Denominator	Conditional proportion	**Relative weight**^**¤**^	Cluster total weight*	**Proportional contribution (total = 0.54)**^**∞**^
**Delay 1 - home delay**				**1/6**	**0.34**	**63%**

# children whose caregivers did not mention at least one severe symptom	12	f	30%	0.04		

# children with possibly severe or severe symptom who were treated at home	28	d	80%	0.11		

# children only receiving treatment at home without going outside for care	5	f	13%	0.02		

# children with severe symptoms who were brought outside the home for care after > 1 day	23	c	82%	0.12		

# children who only received informal health care for their fatal illnesses as both first and last source of care	3	b	12%	0.02		

# not going for referral because of caretaker decision-making	3	e	20%	0.03		

**Delay 2 - transport delay**				**1/2**	**0.06**	**11%**

# delaying > 2 hrs to reach first or last provider	1	a	3%	0.02		

# not going for referral because of lack of money for transport	2	e	13%	0.04		

**Delay 3 - health facility delay**				**1/3**	**0.14**	**26%**

# children obtaining treatment from provider after > 1 hr from first or last provider	5	a	16%	0.05		

# children referred because of lack of equipment or lack of drugs	1	e	7%	0.02		

# deceased children who did not receive any treatment after visiting first or last formal provider	5	b	20%	0.07		

**# who went to at least one outside provider (a)**	32					

**# who went to at more than one outside provider (b)**	25					

**# children reported with at least one severe symptom (c)**	28					

**# children reported with at least one severe or possibly severe symptom (d)**	35					

**# children referred from first or last provider (e)**	15					

**Total # child deaths (f)**	40					

^¤ ^Each indicator's relative contribution to the delay within a cluster, assuming each indicator is equally important.

*The total contribution to delay of each cluster (total = 0.54)

**^∞ ^**The proportional contribution to delay of each cluster.

*Transport delay *was defined as taking more than two hours to reach the first or last provider after a decision to seek care was made or a child not being taken for referral because of a lack of transport or lack of money for transport.

*Health facility delay *was defined as the first or last provider taking more than one hour to attend to the child after the child had reached the health facility, referral to another facility because of lack of equipment or drugs, or the first or last formal care provider not providing any care or treatment.

The relative contribution of delays at home, during transport, or at the health facility was calculated in four steps. 1) First, the conditional proportion of children exposed to delay was calculated by dividing the number of children exposed to each delay indicator by the total number of deceased children who could have been exposed to that delay indicator. 2) Second, each indicator's relative contribution to the delay within a delay cluster (e.g., home, transport, or health facility) was calculated by multiplying the conditional proportions for each delay indicator with the fraction of each delay cluster to avoid over weighting clusters containing more delay indicators. 3) The total contribution to delay of each delay cluster was calculated by summing up the relative weights within each delay cluster. 4) The proportional contribution to delay of the different clusters was calculated by dividing each cluster total by the overall cluster total.

The data from Iganga/Mayuge HDSS and Dodowa HDSS were analyzed independently, and the results are presented separately, as we had no intention of comparing the two different countries but merely wished to present the results of the SA tool in two different contexts.

## Results

### A. Iganga/Mayuge HDSS

#### Child characteristics

A total of 434 deaths in children 29 days old to 5 years old were reported and surveyed between January 2009 and July 2010 in Iganga/Mayuge HDSS, Uganda. The mean age of the deceased children was 17 months (standard deviation [SD]: 13) of whom 48% were female. Forty-two percent (181/434) died at home.

#### Length of illness

Sick children in Iganga/Mayuge HDSS tend to be sick for about one week before they die. The *length of illness *was the time between the mother first noticing that her child was sick and the day of the child's death. The median number of days from first illness symptom was recognized until death occurred was six (interquartile range [IQ]: 2-28).

#### The pathway model

**Figure 1:**
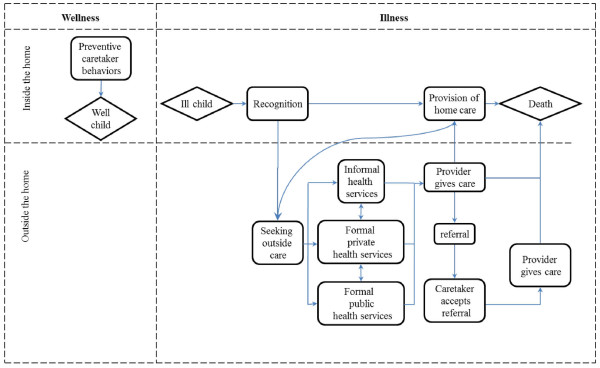
**Conceptual framework of the possible care seeking processes preceding death (modified after Kalter et al. 2004)**.

Using the pathway model (Figure 1), the proportion of caretakers in Iganga/Mayuge HDSS i) recognizing illness and treating at home, ii) seeking outside care from a formal or informal provider, and iii) receiving treatment or referral advice from a provider was calculated.

##### i) Caretakers' recognition of illness and care provided in the home

Although most caretakers (417/434; 96%) recognized that their child was seriously ill, one-third (126/398; 32%) of children with at least one possibly severe or severe symptom was first gave treatment at home. Of these, 85 (20%) did not seek any further care outside the home (Table 1). Sixty-four percent (210/326) of caretakers treated with medicines whereas 23% (74/326) used herbs or traditional remedies. The medicines most commonly used at home were antimalarials (148/210; 70%), paracetamol (89/210; 42%), and antibiotics (48/210; 23%). The most commonly used antimalarials included chloroquine, Coartem (artemether/lumefantrine) and quinine, and the most frequently mentioned antibiotics were Cotrimoxazole (trimethoprim/sulfamethoxazole) and ampicillin. All of the traditional remedies mentioned were harmless, including tea infusions from local leaves, egg yolk, and glucose.

##### ii) Caretakers seeking care outside the home

Overall, 80% (349/434) of the caretakers sought some type of care outside the home for their child's illness, though 9% (28/324; 25 missing values) of these first went to an informal provider (traditional healers or drug shop). Still, more than half (173/324; 53%; 25 missing values) went to a public provider first and 38% (123/324; 25 missing values) went to a formal private provider. Almost half of the 349 caretakers (48%) who sought outside care went to more than one provider and 96% (163/169) went to a public provider for their last source of care. The median time to access a formal health care provider after noticing a severe symptom in the child was three days (IQ: 1-4).

##### iii) Providers giving care

Of the children who were taken outside their home for care, 13% (45/349) did not receive any treatment from the first provider seen, and 10% (17/169) did not receive any treatment from either the first or the last provider. A total of 141 of the 349 children (40%) who were taken to a provider were referred for further treatment, but only half (62/125; 22 missing values) of the caretakers adhered to the referral advice. Among the 62 caretakers who gave a reason for their nonadherence with the referral advice, the vast majority (87%) stated that it was because of a lack of money. Twenty-eight of the caretakers of the deceased children (6%) had been given a death certificate, but none of these stated the cause of death. None of the caretakers who had visited a hospital stated they had been given postmortem results.

#### The three-delay model

**Figure 2:**
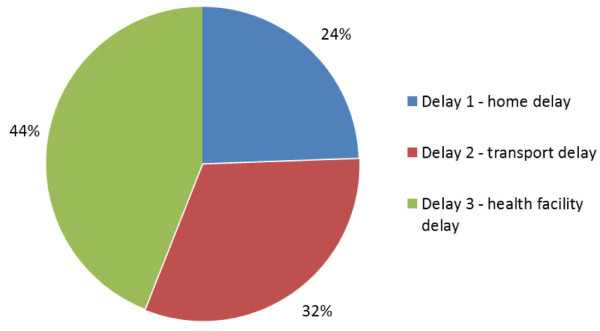
**The relative contribution of the three delays leading up to child death in Iganga/Mayuge HDSS, Uganda (n = 434)**.

The relative contribution of delays at the home, on the way to, or in the health facility was calculated (Table 1). Most delays in the care-seeking process were caused by problems at the health facility (44%) (Figure 2). At the health facility, approximately 20% of children (71/349) were reported to have waited more than one hour to be attended by a health worker, and 65% (92/141) of children who were referred were referred because of lack of drugs or equipment. Seventeen of the 234 caretakers (7%) who went to more than one source of care did not receive any treatment during the care-seeking episode.

The second biggest contribution to delay (33%) was caused by problems with transport. After the caretakers had decided to seek care outside the home, 24% (84/349) spent more than two hours traveling to the health provider. Of the children whose caretakers did not adhere to the referral advice, 12% did so because of lack of money for transport.

Delays in the home contributed to 21% of the total delay. Four percent (17/434) of caretakers did not recognize severe illness symptoms, 32% (126/398) treated severe or possible severe symptoms at home, and 4% (18/434) never took their child for care outside the home. Of the children whose caretakers had recognized the severe symptoms, 42% (174/417) still waited more than one day before they went outside the home to seek treatment. Among the 349 children who were brought outside the home for care, 3 (1%) saw only informal providers during the illness episode. For children who were referred to another provider, 4% (6/141) did not go because of perceptions that the referral was unnecessary or that the child was improving.

### B Dodowa HDSS, Ghana

#### Child characteristics

The analysis was based on the 40 deaths in children 29 days old to 5 years old between December 2008 and December 2009. One child was excluded because he died without having been ill. The mean age of the deceased children was 21.7 months (SD: 15.7), and 43% were females. Forty-three percent (17/40) had died at home.

#### Length of illness

Sick children in Dodowa HDSS tended to die quickly after the illness was recognized. The *length of illness *from the time between the caretakers first noticed that a child was sick and the day of the child's death was only 3.5 days (IQ: 2-6).

#### The pathway model

As for Iganga/Mayuge HDSS, the pathway model (Figure 1) was used to calculate the proportion of caretakers in Dodowa HDSS who i) recognized illness and treated the child at home, ii) sought outside care from formal or informal providers and iii) received treatment or referral advice from a provider.

##### i) Caretakers' recognition of illness and care provided in the home

Of the 40 caretakers interviewed, 28 (70%) mentioned having seen at least one severe symptom in the child. Of these, five (18%) only gave treatment at home without going for outside care whereas three died without receiving any kind of treatment. Most caretakers (28/40; 70%) first gave herbal or orthodox treatments or both at home before seeking care outside. Half of the caretakers gave drugs bought from drug shops, 36% (10/28) gave herbs, and 14% (4/28) gave a combination of orthodox and herbal medicines. A mix of paracetamol, antimalarials, and antibiotics were the most frequently mentioned medicines used in the home. The traditional medicines included an unspecified mix of leaves, herbal teas, and herbs for bathing the child.

##### ii) Caretakers seeking care outside the home

Overall, 80% (32/40) of caretakers sought care outside the home for the child's illness, though 44% (14/32) first went to informal health providers like traditional healers and drug shops. Nineteen percent (6/32) first went to formal private providers, and 38% (12/32) went to a public provider. Most of the 32 caretakers (78%) who sought care outside the home went to more than one provider, and 56% (18/32) went to a public provider as the last source of care.

Still, 38% (12/32) went to a public provider first, and 19% (6/32) went to a formal private provider. The majority of the 32 caretakers (78%) who sought outside care went to more than one provider, and 56% (18/32) went to a public provider for their last source of care. The median time to access a formal health care provider after noticing a severe symptom in the child was two days (IQ: 1-5).

##### iii) Provider giving care

Of the children who were taken outside the home for care, 16% (5/32) did not receive any treatment from the first provider, and 20% (5/25) did not receive any treatment from the last provider. A total of 15 of the 32 children (47%) who were taken outside the home for care were referred for further treatment, but only nine (60%) of the caretakers adhered to the referral advice. The reasons for nonadherence with the referral advice was the death of the child before reaching the referral point (4 children), delayed caretaker decision making (3 children) and financial barriers hampering care seeking (2 children). Three caretakers (7.5%) stated they had received a death certificate, and only five caretakers received a postmortem result. However, the certificates were not with the respondents at the time of the interview (the document could not be found, was with someone else, or was kept at the hospital).

#### The three delay model

**Figure 3:**
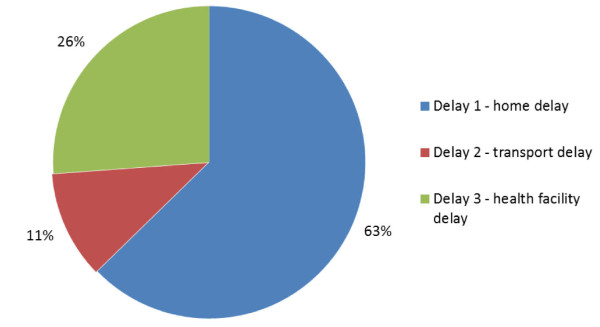
**The relative contribution of the three delays leading up to child death in Dodowa HDSS, Ghana (n = 40)**.

The relative contribution of delays at the home, on the way to, or in the health facility was calculated (Table 2). Most delays (63%) were caused by factors in the household, where the main barriers were poor caretaker recognition of severe illness and delayed decision-making to seek formal care outside the house for the sick child (Figure 3). Thirty percent (12/40) of caretakers did not mention any severe symptoms seen in the child before death, and all caretakers who recognized severe or possibly severe symptoms first treated at home. Of the caretakers who recognized that their children had severe symptoms, 82% (23/28) still waited more than one day before they took the child outside the home for treatment.

The second biggest delay was caused by problems in the health facility (26%). At the health care provider, 16% of children (5/32) were reported to have waited more than one hour to be attended by a health care worker, and 20% (5/25) did not receive any treatment from either the first or last health care provider.

The smallest contribution to delay (11%) was caused by problems with transport. Of the 32 caretakers who decided to seek care outside the home, all but 1 spent less than two hours traveling to the health provider, while one could not tell the actual time she spent travelling. Two of the caretakers who did not adhere with referral advice did not go because of lack of money for transport.

## Discussion

The SA questionnaire for deceased children under 5 years old developed by the INDEPTH SAWG was found to be useful for quantifying contributing factors to child death at the health facility, community, and household levels. In our study, we found that lack of symptom recognition and household practices (delay 1) was the most common cause of delay in Dodowa HDSS, while health facility-related delays (delay 3) were the primary reasons for delay in Iganga/Mayuge HDSS in Uganda.

Our results are consistent with earlier studies of the possible contribution of inadequate care seeking and poor case management to childhood deaths [10,11,19]. An ethnographic study in Ghana found that mothers may not be able to recognize serious illness in their babies, and they often do not seek care outside the home even when they do realize that their child is seriously ill [29]. Studies in older children conducted in the same setting [30,31] and elsewhere in Uganda [32] have shown similar challenges in care seeking and referral. Other barriers, which could not be elucidated by the present semistructured social autopsy tool, include gender aspects of decision-making, other responsibilities at home, local perceptions of illness and care providers, poverty, and distance to health facilities [30,31,33-35]. Which of these barriers led to delays at home cannot be explained by the social autopsy. However, other studies have previously shown that it is usually a combination of factors, of which poverty and distance to health centers are key determinants for late care seeking [30]. To improve policies and programs, it is critical for local health authorities to understand cultural issues and social beliefs, and we recommend that program implementers complement SA investigation with in-depth qualitative methods for a sample of cases (e.g., using case narratives with probing).

Although most sick children were taken to qualified providers at some stage, many caretakers waited more than 24 hours after illness recognition before seeking any care outside the home. A median of two and three days passed between a caretaker noticing a severe symptom and seeking care from a formal health practitioner in Dodowa and in Iganga/Mayuge, respectively. Waiting for self-prescribed medicines to have an effect is one likely explanation for some of the later attendance at formal providers. Having used antibiotics in the home has previously been shown to be the only significant risk factor for late care seeking in children who later died of pneumonia in Uganda [19].

Our findings are also in accordance with other studies on fatal childhood illnesses in Guinea-Bissau, Tanzania, South Africa, and India, which showed high attendance at health facilities before child death [9,12,16,18]. Care in district hospitals is of poor quality in both Ghana and Uganda [36,37], and the fatal outcome of Ugandan children, who in a majority of cases had seen a formal health care provider prior to death, is likely explained by a combination of factors, such as late arrival of very sick children and district hospital incapacity to cater to critically ill children. Yet even though caretakers are aware of the poor quality of care in the public health system [31], they often have no choice when circumstances become grave. They will seek care there anyway, even though they know that treatment and staff may not be available. Hence, the quality of primary health care providers, both private and public, cannot be overlooked, and training of health workers is needed in sick child case management and in caretaker education on symptom recognition.

The social autopsy approach provided descriptive information on fatal cases that is not routinely available, such as the timing of the events, the place of death, caretaker behavior during the illness episode, the number and type of health professionals involved, and details of treatments and advice given. Elaborating this sequence is essential to understanding the factors and constraints external to the disease itself that may be associated with the childhood deaths and that must be addressed when designing, implementing, and monitoring intervention strategies to reduce childhood deaths, such as the IMCI initiative or community case management programs for sick children.

One challenge faced by sites that implemented the SA tool in this study was how to integrate the SA tool into the VA tool without creating an instrument that was overly cumbersome. Collecting the SA data separately from the VA data during different household visits may shorten the time for the SA interview but would negatively affect the flow of questions, as many VA and SA indicators are interlinked and difficult to separate (e.g., timing of care seeking in relation to symptoms mentioned).

Another challenge was the complexity of analyzing care-seeking data. Following the advice provided by Aguilar et al. (1998) [20] and learning from the experiences from Bandim HDSS in Guinea-Bissau, where caretakers had a difficult time remembering the order in which different providers were seen (Personal Communication with Rikke Thoft Nielsen 2011), the tool was designed to only capture information on the first and last providers. While this may exclude valuable information that could explain behavior, it was deemed a necessary revision of the tool to reduce the complexity for the data collectors and analysts to a manageable level. To overcome this complexity, developing a computerized coding of SA data as is done for verbal autopsy [2,38] would be preferable. With more programming, these tools could potentially adopt the structure of the Tanzania Essential Health Interventions Project (TEHIP) District Health Intervention Profile, which not only displays the local disease burden but also links the data to displays of health systems actions that can be implemented to address the most common problems and barriers [39]. Another option used in other studies [10,11] is to have an expert panel review each death using standard checklists and frameworks, which is a model commonly used for the interpretation of VA data.

This study has some limitations. The illness history and care-seeking information are based on interviews by nonmedical personnel. Interviews depending on recall pose reliability and validity problems [40]. However, severe symptoms are normally remembered longer than mild symptoms [41]. Since most interviews were made within four to six weeks of death, recall bias was likely low. In these retrospective interviews we were not able to determine the actual quality of care provided to these children, nor were we able to adequately determine social and cultural processes in the family that affected the care seeking. Other methods (e.g., clinical audits and in-depth interviews) will be needed to investigate these factors. Another important limitation was the failure to analyze the sequence of symptoms in the order that they appeared and, hence, the care-seeking patterns in relation to the severity of the disease. Given the importance of illness severity as a parameter in the analysis, a question on alertness, activeness, and feeding practices was added in the final tool to assess the status of the child at the time of illness recognition and care seeking. As we collected information only on children that died, we cannot conclude whether any of the delays had a negative effect on survival, as this would require a case-control approach. However, some practices reported, such as mothers only seeking outside care when the child was severely sick or severely sick children not receiving any treatment for the illness after having seen one or more providers, are likely to be detrimental to survival of sick children.

## Conclusions

Our team has shown that social autopsy data can be collected as part of verbal autopsy data and that such data could be useful for informing the design, implementation, and monitoring of interventions. It is often perceived that the key challenge to effective coverage of child survival interventions is poor caretaker care-seeking behavior. Our findings reveal that while delays at home were indeed common in Dodowa HDSS in Ghana, a main challenge in Iganga/Mayuge HDSS in Uganda was the sick child case management in the health system. Meanwhile, caretaker awareness of danger signs needs to improve in both countries to promote early care seeking and reduce the number of children needing referral. Social autopsy is a promising method to improve the understanding of the circumstances preceding death. Access to this information can help health planners and policymakers prioritize scarce resources appropriately by identifying the most suitable interventions for the specific context.

## Competing interests

The authors declare that they have no competing interests.

## Authors' contributions

KK and DK were involved in the conception and design of this work, the data collection, the analysis and interpretation of the data, and in the writing of the manuscript.

LY, RTN, and AM were involved in the conception and design of this work, data collection, and in the writing of the manuscript.

JA and CN were involved in data collection, data analysis, and in the writing of the manuscript.

TNW, MG, AA, and PW were involved in the conception and design of this work and in the writing of the manuscript.

All authors read and approved the final manuscript.
